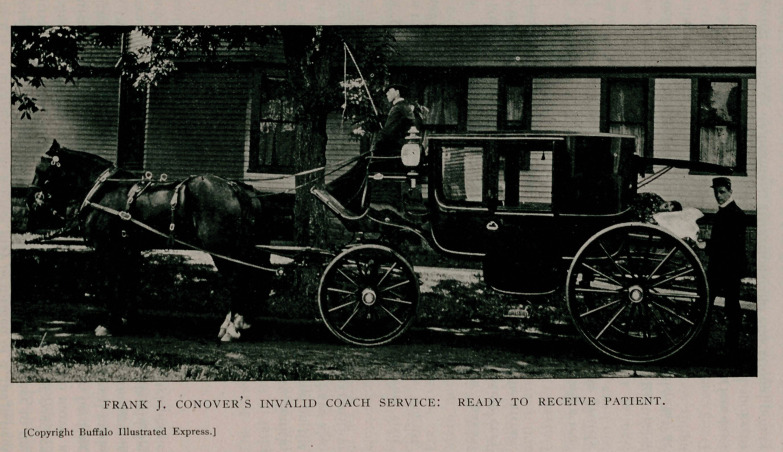# American Public Health Association

**Published:** 1901-09

**Authors:** 


### American Public Health Association.

THE American Public Health Association will hold its 29th annual meeting at Buffalo, September 16-21, 1901, as announced elsewhere. It will be remembered that this great medical organisation, which represents the United States, Canada, and Mexico, held a meeting here five years ago, under the presidency of Eduardo Liceaga of Mexico The memory of that splendid meeting is still fresh and, great as it was, the forthcoming meeting should be still greater not only in numbers, but also in work accomplished.

It is one of the most important medical bodies in existence and is also one of the largest in its representation. Every health board in the three countries mentioned sends delegates to its meetings and all health officers, public health officials, sanitarians sanitary engineers, and many publicists are members. It carries on its rolls some of the most distinguished names known in medicine—indeed, in science—in the world, and its prospective Buffalo meeting should be made the greatest medical congress ever held in the City of the Exposition.

The men and women who will attend it are engaged in studying the greatest problem that concerns humanity today—namely, the prevention of disease. This is the chief concern of the educated physician of this period, and to its solution many great men are devoting their lives and their purses. Every business man is or ought to be interested in this subject, and every man and woman of intelligence in Buffalo ought to feel an interest in the success of the September meeting here.

Pecuniary support is needed to carry such a meeting forward to a successful conclusion. A large number of visitors will come to the Pan-American City in consequence of this gathering. They should be entertained in a manner commensurate with the importance of their errand,and in keeping with the distinguished character of the visitors. High public officials, noted citizens, great officers of the Army and Navy and Marine Hospital Service, and distinguished foreign representatives will be here, and they should be welcomed with that hospitality for which the Queen City of the Lakes is so justly celebrated.

Contributions of money should be liberal from our citizens in general, and such may be sent to Dr. Henry Reed Hopkins, chairman of the committee of arrangements, to Dr. D. W. Harrington, chairman of the committee on finance, or to Dr. Ernest Wende, chairman of committee on membership. An acknowledgement will be promptly made and the amounts properly credited.

---

Dr. Nelson W. Wilson, sanitary officer of the exposition is kept busy in looking after the health of the Rainbow City. When one considers that the exposition is really a vast camp being occupied for six months, inhabited by thousands of people who permanently reside within its lines, and which is visited by millions of others who are coming and going constantly and who must be fed and cared for in various ways, some conception may be formed of the duties of the sanitary officer. He must be on the alert to detect any infraction of the rules and he must enforce his orders with an imperial hand. That no serious sickness has disturbed the inhabitants of the Midway, which numbers amongst its population people of all climes and of every race and nation on the face of the globe, many of whom have been indifferently trained and some being entirely ignorant of sanitary laws, it seems almost marvelous that no epidemic or infectious disease has appeared.

Sometimes it has been necessary to enforce orders with apparent severity, but every concessionaire and exhibitor will recognise that he has no better friend than a sanitary officer who performs his duties in a fearless manner.

To show how alert one must be, several days ago the sanitary officer seized several phosphate bottles with metal tops, and destroyed them. They were being used by two independent drink-stand concessionaires. Only glass and porcelain tops are allowed on phosphate bottles, because the gases of the phosphate corrode the metal and breed disease. Dr. Wilson inspects daily all drink-dispensing and food-selling booths, and it is asserted that there is not one thing to eat or drink sold on the grounds that is in any way unclean, if rigid inspection can prevent it.

---

The *Medical Mirror*, in its issue for July, 1901, does the Journal great honor in republishing several columns of its editorial work. We appreciate the compliment because there is no better judge of the value of medical journal articles, editorial and otherwise, than the veteran and scholarly editor of the *Mirror*.

Dr. Love thinks it would have been better if the reorganisation scheme of the American Medical Association had been laid over for a year, rather than that action were taken at the late meeting. Dr. Love makes the mistake—we trust we may be pardoned for terming it such—of speaking as though it were an amendment that was acted upon instead of a plan of reorganization. The two conditions are entirely different. The house, at Atlantic City, instructed a committee to report a scheme of reorganisation. After a year's work the committee so reported at St. Paul. It reported to an entirely different house than that which created it. The personnel of the house changes every year. This has been a great drawback to intelligent action—an evil which is now corrected. The action at St. Paul was taken with deliberation and every section of the plan received seriatim consideration and action. Had action been delayed for a year the committee would have reported to an entirely new house with new officers, who would have known less of the affairs under consideration than those in authority this year. It seems to us that the only logical action the house could take was to approve or reject the scheme of the committee. It did the latter with some amendments, and 'tis well.

---

An Invalid Coach Service.—Mr. Frank J. Conover, of 182-186 Herkimer Street, Buffalo, offers the public a service which will be highly appreciated in those cases of accident or sudden sickness, where transportation is desired without the publicity of the common ambulance. This he offers through R. R. Bennett's "invalid coach," view of which is shown on the opposite page.

This coach was built for the purpose of conveying incapacitated persons free from the annoyance incident to transit in an ambulance. It is of the Berlin style, provided with an air mattress and pillow, the bed being fully six feet in length. On the inside it will accommodate two persons with the patient. The wheels are supplied with rubber tire and the removal is accomplished noiselessly and without the jar of an ordinary vehicle. After the patient is in the carriage, and the back closed, there is nothing to indicate its character—to all eyes it is simply one of the many fine carriages that pass through the streets. When a patient is to be brought into town, if the distance is too far to send the

FRANK J. CONOVER'S INVALID COACH SERVICE: READY TO RECEIVE PATIENT.

[Copyright Buffalo Illustrated Express.]

carriage, one or two experienced men are sent with the bed, which is so arranged that a litter may be formed of it, the patient remaining on it aboard the train and is transferred thence to the carriage without change. A special rate is made for coaches going out of town. In cases where it is desired to move a patient from one room to another in the house, it can be done without distress to the individual, by having the removal made by two careful men. This coach will not be used for contagious diseases. For rates and all particulars, application should be made to Mr. Conover, at the above address. His telephone number is Bryant 453.

## Figures and Tables

**Figure f1:**